# Bidirectional crosstalk between PD-L1 expression and epithelial to mesenchymal transition: Significance in claudin-low breast cancer cells

**DOI:** 10.1186/s12943-015-0421-2

**Published:** 2015-08-07

**Authors:** Abdullah Alsuliman, Dilek Colak, Olfat Al-Harazi, Hanaa Fitwi, Asma Tulbah, Taher Al-Tweigeri, Monther Al-Alwan, Hazem Ghebeh

**Affiliations:** Stem Cell & Tissue Re-engineering Program, King Faisal Specialist Hospital and Research Centre, Riyadh, Saudi Arabia; Department of Biostatistics, Epidemiology and Scientific Computing, King Faisal Specialist Hospital and Research Centre, Riyadh, Saudi Arabia; Department of Pathology and Laboratory Medicine, King Faisal Specialist Hospital and Research Centre, Riyadh, Saudi Arabia; Oncology Centre, King Faisal Specialist Hospital and Research Centre, Riyadh, Saudi Arabia; College of Medicine, Al-Faisal University, Riyadh, Saudi Arabia

**Keywords:** CD274, Epithelial to Mesenchymal Transition, Claudin-low, PI3K, TGF-β1, Breast Cancer

## Abstract

**Background:**

The T-cell inhibitory molecule PD-L1 (B7-H1, CD274) is expressed on tumor cells of a subset of breast cancer patients. However, the mechanism that regulates PD-L1 expression in this group of patients is still not well-identified.

**Methods:**

We have used loss and gain of function gene manipulation approach, multi-parametric flow cytometry, large scale gene expression dataset analysis and immunohistochemistry of breast cancer tissue sections.

**Results:**

Induction of epithelial to mesenchymal transition (EMT) in human mammary epithelial cells upregulated PD-L1 expression, which was dependent mainly on the activation of the PI3K/AKT pathway. Interestingly, gene expression signatures available from large cohort of breast tumors showed a significant correlation between EMT score and the PD-L1 mRNA level (p < 0.001). Strikingly, very strong association (p < 0.0001) was found between PD-L1 expression and claudin-low subset of breast cancer, which is known to have high EMT score. On the protein level, significant correlation was found between PD-L1 expression and standard markers of EMT (*p* = 0.005) in 67 breast cancer patients. Importantly, specific downregulation of PD-L1 in claudin-low breast cancer cells showed signs of EMT reversal as manifested by CD44 and Vimentin downregulation and CD24 upregulation.

**Conclusions:**

We have demonstrated a bidirectional effect between EMT status and PD-L1 expression especially in claudin-low subtype of breast cancer cells. Our findings highlights the potential dual benefit of anti-PD-L1 particularly in this subset of breast cancer patients that will likely benefit more from anti-PD-L1 targeted therapy as well as in monitoring biological changes upon treatment.

**Electronic supplementary material:**

The online version of this article (doi:10.1186/s12943-015-0421-2) contains supplementary material, which is available to authorized users.

## Background

Breast cancer is the most common cancer affecting women [1]. In the past, the management of this disease has improved greatly upon patients’ segregation based on their estrogen and progesterone receptors expression [2] and later on by their her2/neu over-expression status [3]. Recently, the remaining tumors that are negative for estrogen receptors, progesterone receptors and Her/neu overexpression are further classified into subtypes (like basal-like and claudin low) and there is evidence that different subtypes respond differently to various treatment regimens [4, 5]. Further investigations are needed to develop new therapies for these emerging subtypes of breast cancer.

PD-L1 (also called B7-H1 and CD274) is a T cell inhibitory molecule that is expressed on antigen presenting cells (APC) leading to T cell anergy and/or apoptosis upon ligation to its receptor programmed death-1 (PD-1, CD279) on T cells [6, 7]. PD-L1 was found to be aberrantly over-expressed in several malignancies where a strong link between its expression in cancer cells and patients’ clinicopathological status has been demonstrated (Reviewed in [8]). Importantly, in many cancer types PD-L1 expression is associated with worse outcome, suggesting the contribution of PD-L1 to promote tumor escape from the immune system [9–12].

We and other have previously demonstrated that PD-L1 expression is associated with triple negative breast cancer [10, 13–15]. In a subsequent complementary study, we have shown that tumoral expression of PD-L1 was accompanied by the infiltration of regulatory T-cells (T-reg), a subpopulation of T-cells known to inhibit the proliferation and cytokine production of effector T-cells [16]. Therefore, PD-L1 and T-reg can provide an immune suppressive microenvironment, leading to tumor escape from immune recognition and promotes tumor growth.

Several mechanisms that regulate PD-L1 expression in cancer cells have been described, including the presence of inflammatory mediators like the INF-γ [17] within the tumor microenvironment, the loss of phosphatase and tensin homologue (PTEN) with consequent phosphatidylinositol-3-OH kinase (PI3K) activation [18], and promotion of cancer cell proliferation [19]. Nevertheless, many breast cancer cells *in vivo* and *in vitro* constitutively express PD-L1 despite normal PTEN and in the lack of INF-γ, suggesting that another mechanism may be involved in regulating this process in breast cancer cells. Understanding the mechanism of PD-L1 induction can help in reversing it upon therapy.

Anti-PD-L1 antibody targeting therapy is currently available and has been shown to be safe and effective in inducing complete or partial response in several carcinomas [20, 21]. Whether anti-PD-L1 blocking antibodies will be effective in breast cancer or not is not yet known. However, before trying this therapy the subset of breast cancer patients who will likely upregulates this molecule and the mechanism of this upregulation should be identified.

In this paper, we have demonstrated that PD-L1 expression is induced upon EMT process and is highly significantly associated with claudin-low, a subtype of breast cancer, known to have EMT features. Importantly, downregulating PD-L1 reversed EMT process in a claudin-low breast cancer cells, strongly suggesting an important role for PD-L1 targeted therapy in this subset of breast cancer.

## Methods and materials

### Patient selection and consenting

This study was conducted in accordance with the Helsinki Declaration and it was approved by the Research Advisory Council (RAC# 2140–001) of King Faisal Specialist Hospital and Research Centre (KFSH&RC). Normal human mammary gland tissues were obtained from 2 patients admitted to KFSH&RC who underwent reduction mammoplasty with no previous history of breast cancer. Sections from Archived paraffin embedded Breast cancer samples were obtained from 67 patients diagnosed with invasive ductal carcinoma of the breast and were previously described [13, 16]. All patients signed an informed consent approved by KFSH&RC.

### Normal breast tissue processing and cell preparation

Normal breast tissues were digested as previously described [19, 22]. Briefly, tissues from reduction mammoplasty) were digested in collagenase digestion medium (Stem Cell Technologies (SCT), Vancouver, Canada) and agitated at 37 °C overnight and cell suspensions were centrifuged at 800 g for 8 min at 4 °C. Cells were differentially centrifuged at 120 g for 2 min at 4 °C to enrich for epithelial cells. The epithelial-enriched pellet was further digested with accutase (SCT) at 37 °C (30–60 min) and were cultured as previously described [23]. The supernatant (mesenchymal-enriched fraction) were centrifuged at 450 g for 8 min, then cultured.

### Cell culture, transfection and selection

The Breast cancer cell lines MDA-MB-231 and MCF-7 (ATCC) were maintained in DMEM (Sigma) supplemented with fetal bovine serum (FBS, Invitrogen) and antibiotics and antimycotics (ABM, Invitrogen). Normal breast cell lines: MCF-12A and MCF10A (ATCC) were cultured in universal medium composed of DMEM/F12 medium supplemented with 10 μg/mL insulin, 20 ng/mL epidermal growth factor (EGF), 500 ng/mL hydrocortisone (all from Sigma) and 100 ng/mL cholera Toxin (LIST Biological Laboratories, Surrey, United Kingdom), 5 % horse serum (Invitrogen) and 1 % ABM. All established cell lines were used within 6 months of purchase from ATCC.

Primary cells were selected from overnight digested normal mammary tissue (see above). Primary luminal cells were further isolated based on their Ep-CAM positivity using MACS (Miltenyi Biotec, Germany) cell separation system. HMLE cell line were generated by transecting primary luminal cells at passage 1–3 sequentially with lentiviruses expressing SV40 (small and long T antigen) and h-tert (both from ABMgood, Canada). Both primary luminal cells and immortalized luminal cells (HMLE) were propagated in serum free WIT-P medium (Stemgent, Cambridge, USA) while primary mesenchymal cells were cultured in DMEM/F12 supplemented with 10 % FBS.

PD-L1 were downregulated using specific Sh-RNA to PD-L1 (in a retroviral pGFP-V-RS vector from Origene). Other specific Sh-RNA were used also for confirmation (in a lentiviral TRIPZ commercially available vector, which is activated only in the presence of doxycycline (1 μg/mL). Selection for PD-L1 Sh-RNA (Sh-PD-L1) transfected cells were made using puromycin (1 μg/mL). PD-L1 expression in T-47D was induced by transfecting cells with PD-L1 ORF (pCMV6-AC-GFP vector from Origene) and the selection for PD-L1 ORF transfected cells were made using G418 (500 μg/mL).

EMT was induced using TGF-β1 (2.5 ng/mL, R&D systems) with daily medium exchange using freshly thawed TGF-β 1 aliquots. TNF-α, IL-6 and EGF were used at a concentration of 100 ng/mL. Major pathways involved in EMT induction were tested on MCF-12A cells using the general PI3K/AKT pathway inhibitor LY294002 (20 μM), the MEK/ERK inhibitor U0126 (10 μM), the SMAD inhibitor SiS3 (5 μM) and the NF-kB inhibitor Bay 11-7082 (2.5 μM), all from Millipore.

### Flow cytometry

Cells were incubated for 30 min on ice with directly labeled antibodies. Cells were then washed with PBS followed by fixation in 0.5 % paraformaldehyde in PBS. Data were acquired using an LSR II flow cytometer using BD FACSDiva operating software. Positive staining was considered based on the negativity of an isotype control. A minimum of 10,000 events were recorded for all samples.

### Immunohistochemistry

Immunohistochemistry for vimentin and E-cadherin were done on formalin-fixed, paraffin-embedded tissue of previously described breast cancer samples [13, 16]. Vimentin was detected using ready-to-use mouse anti-human vimentin antibody from Ventana (Cat# 790-2917) and were stained using automated staining platform (Ventana). Immunohistochemical staining of E-cadherin was done manually using rabbit anti E-cadherin antibody (clone EP00y) overnight at a dilution of 1:500. Envision + polymer (ready to use; Dako) was used as a secondary antibody. Color was developed with 3,3′-diaminobenzidine (DAB) and instant hematoxylin (Shandon) was used for counterstaining.

### Expression analysis of human breast tumor microarray datasets

The first set of samples were from GSE18864 (*n* = 84) that contains gene expression data from 84 breast cancer patients, 38 of which were triple negative breast cancer (TNBC), obtained using Affymetrix’s one-channel microarray, namely, GeneChip® Human Genome U133 Plus 2.0 Arrays. The data was downloaded from Gene Expression Omnibus (GEO; http://www.ncbi.nlm.nih.gov/geo/). The second set of samples analyzed consisted of mRNA expression profiling of breast invasive carcinoma samples (*n* = 530) performed using Agilent G450A_07 arrays from Cancer Genome Atlas (TCGA) project (http://cancergenome.nih.gov) [24], and TCGA level 3, the most highly processed data were downloaded in accordance with TCGA Data Access Policies (https://tcga-data.nci.nih.gov/tcga/).

For each dataset, an EMT score for each sample was calculated as the average expression of genes up-regulated in EMT minus the average expression of genes down-regulated in EMT using the 17 EMT signature genes (ZEB1, ZEB2, Snai1, Snail2, Twist1, Twist2, FOXC2, VIM, FN1, SOX10, MMP2, MMP3, CDH1, CLDN3, CLDN4, CLDN7 and DSP). The claudin-low (CL) scores for each sample were calculated based on mRNA expression of claudin-low gene signature (1604 genes from Prat el [5]). CL score was calculated as the average expression of genes up-regulated in CL subtype of breast cancer minus that are down-regulated in CL subtype.

### Statistical analysis

Significance in expression or mammosphere formation was determined by Student’s t-test. Comparison of categories within a given characteristic was carried out with the χ^2^ test, if any of the expected frequencies was less than five, the Fisher’s exact test was used. Two-sided *P*-value of < 0.05 was considered statistically significant. Statistical analyses were performed using MATLAB software packages (Mathworks, Natick, MA, USA), JMP® Pro11, GraphPad Software, Inc and PARTEK Genomics Suite (Partek Inc, St. Lois, MO, USA).

### Results

#### PD-L1 expression level is associated with the mesenchymal features of normal and malignant breast

**Fig. 1:**
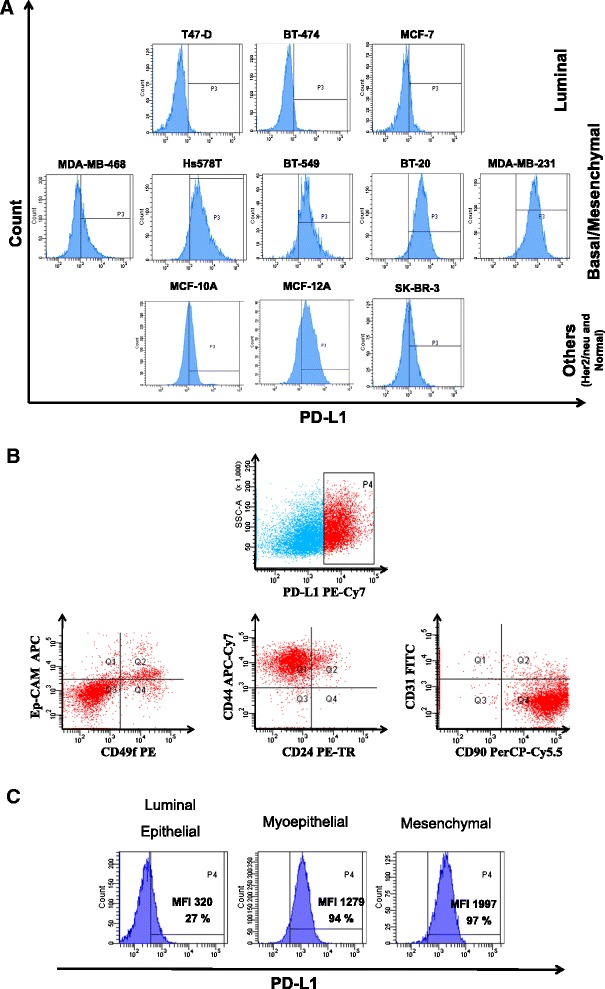
PD-L1 is associated with mesenchymal status of normal and malignant breast. **a** Normal and breast cancer established cell lines grouped into luminal-like, basal/mesenchymal-like breast cancer cells and others including normal breast cell lines. **b** Representative flow cytometry dot plots showing the phenotype of PD-L1 positive segregated normal human breast cells as measured by Ep-CAM/CD49f, CD44/CD24 and CD90/CD31 expression and as analyzed by multi-parametric flow cytometry following serial gating to remove debris, dead and hematopoietic cells. **c** Representative flow cytometry histogram showing the level of PD-L1 expression in primary cultured epithelial (luminal and myoepithelial) and mesenchymal cells

To understand the mechanism that regulates PD-L1 constitutive expression in some native PTEN expressing breast cancer cells and in the absence of INF-γ we screened breast cancer cell lines available in our laboratory according to their level of PD-L1 expression. Most of the PD-L1 positive cell lines had mesenchymal features, while PD-L1 negative cell lines demonstrated luminal characteristics (Fig. 1a). The correlation between PD-L1 expression and mesenchymal features in breast cancer cell lines prompted us to investigate if such relationship is physiologically relevant to the normal human breast. Therefore, we determined the level of PD-L1 in the different subpopulations of unmanipulated, uncultured segregated human mammary gland cells. Using multi-parametric flow cytometry, which we have previously established [22], PD-L1 positive cells could be identified as Ep-CAM^neg^/CD49f^neg^, CD44^high^/CD24^low^, CD90^high^/CD31^neg^, a typical phenotype for the mesenchymal fraction of human breast cells (Fig. 1b, detailed gating is available in Additional file 1: Figure S1). We have further isolated the epithelial (luminal and myoepithelial) and mesenchymal fractions of the human breast cells and cultured them *in vitro* for 1–2 passages. In consistence with their *in vivo* phenotype, mesenchymal cells in culture (*in vitro*) had the highest level of PD-L1 expression, while luminal cells had the lowest level of expression (Fig. 1c). The above data show an association between mesenchymal features and the level of PD-L1 expression in normal human breast cells. Together, our data showed that PD-L1 expression level is associated with the mesenchymal features of normal and malignant breast cells.

**Fig. 2:**
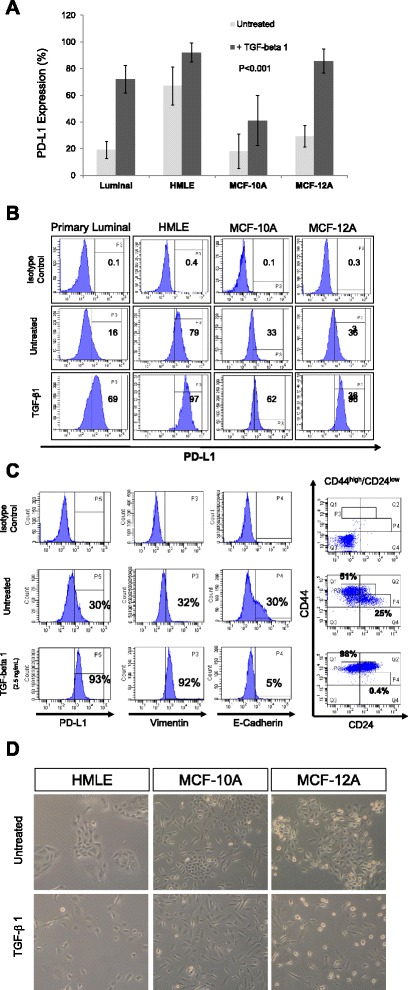
Epithelial to mesenchymal transition (EMT) upregulates PD-L1 in the human breast cells. **a** Bar graphs showing the expression level of PD-L1 as measured by flow cytometry in four normal breast cells before and after EMT induction using continuous treatment with TGF-β1 (2.5 ng/mL) for 7 days for MCF-12A and MCF10A cells and 14 days for primary luminal and HMLE cells. Bars represent the means of 3 independent experiments ± standard deviation (SD) **b** Representative flow cytometry histograms for PD-L1 and **c** a typical image for cells before and after EMT induction. **d** representative flow cytometry histograms showing the expression of intracellular vimentin/E-cadherin molecules and dot blots of CD44/CD24 cell surface markers of MCF12A after EMT induction using TGF-β1 (2.5 ng/mL)

In order to demonstrate the effect of mesenchymal status on PD-L1 expression, we induced epithelial to mesenchymal transition (EMT) using continuous TGF-β1 treatment on three normal cell lines (HMLE, MCF10A and MCF12A) and a primary luminal epithelial cells and measured PD-L1 protein expression using flow cytometry. Induction of EMT reproducibly and significantly upregulated PD-L1 up to 3 folds in all tested normal cells (Fig. 2a & b, p < 0.001). EMT-mediated PD-L1 upregulation was parallel to upregulation of CD44 and the down regulation of CD24 surface markers (Fig. 2c) and morphological changes from cobble-stone tightly arranged cells (epithelial-like) to spindle shaped widely distributed (mesenchymal-like) cells (Fig. 2d). The effect of TGF-β1 induced PD-L1 was not limited to normal breast cells as breast cancer cell lines also showed similar results albeit with much less efficiency than normal cells (Additional file 1: Figure S2A).

To confirm that the upregulation of PD-L1 expression is due to the EMT process and not TGF-β1 itself, we induced EMT by transfection with H-RAS oncogene, or treatment with IL-6, TNF-α, or EGF. Transfection of MCF-12A normal breast cells with H-RAS upregulated PD-L1 expression up to 2 folds. Similarly treatment with IL-6, TNF-α, and EGF upregulated PD-L1 (Additional file 1: Figure S2B). The above data show that the EMT process upregulates PD-L1 expression in normal and malignant human breast epithelial cells.

#### Upregulation of PD-L1 is dependent on the PI3K/AKT and MEK/ERK pathways activation

**Fig. 3:**
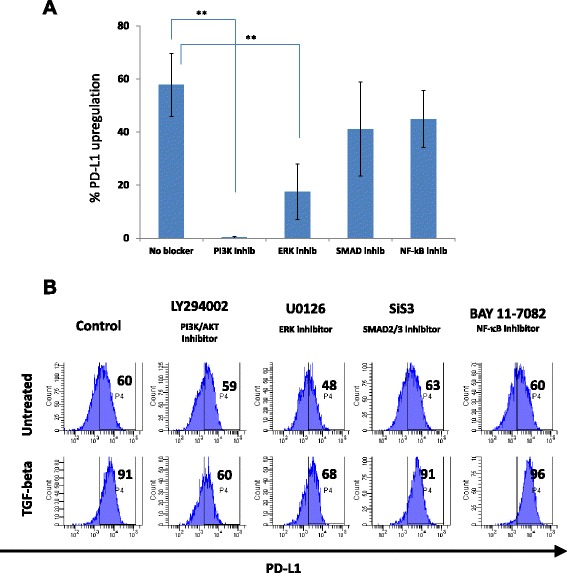
PI3K and ERK inhibitors abrogates EMT-mediated PD-L1 upregulation **a** EMT-mediated PD-L1 upregulation in MCF-12A cells in the presence or absence of major EMT pathway inhibitors, calculated as a percentage of the level of PD-L1 in untreated cells as per the equation $\%\ \mathrm{upregulation}=\frac{\mathrm{PDL}1\ \mathrm{expression}\ \mathrm{after}\ \mathrm{T}\mathrm{G}\mathrm{F}1\ \mathrm{treatment}-\mathrm{P}\mathrm{D}\mathrm{L}1\ \mathrm{before}\ \mathrm{T}\mathrm{G}\mathrm{F}1\ \mathrm{treatment}}{\mathrm{PDL}1\ \mathrm{before}\ \mathrm{T}\mathrm{G}\mathrm{F}1\ \mathrm{treatment}\ \mathrm{and}\ \mathrm{without}\ \mathrm{any}\ \mathrm{blocker}} \times 100$ **b** Representative flow cytometry histogram of PD-L1 before and after TGF-β1 treatment in the presence or absence of EMT inhibitors

To understand the mechanism of EMT-mediated PD-L1 upregulation in breast cells, we have used MCF-12A normal breast cells as they were the most efficient in undergoing an EMT. We exposed MCF-12A to continuous TGF-β1 treatment in the presence or absence of specific inhibitors of the 4 main pathways known to be involved in EMT induction [25, 26]. Among all tested PI3K, ERK, SMAD, and NF-κB pathway blockers, the PI3K/AKT pathway inhibitor (LY294002) was the most successful in abolishing the up-regulation of PD-L1 protein expression upon EMT induction as measured by flow cytometry (Fig. 3a), and to a lesser extent MEK/ERK inhibitor (U0126). On the other hand, NF-κB blocker (Bay 11–8072) and SMAD 2/3 inhibitor (SiS3) had no or very minor effect on PD-L1 upregulation (Fig. 3a & b).

#### Gene expression signatures (GES) show tight association between PD-L1, EMT and claudin low breast cancer

To expand our *in vitro* findings and to test the association of EMT status with PD-L1 mRNA expression in breast cancer patients, we used large number of breast cancer patients’ gene expression profiling data from publically available databases. We calculated the EMT score of each breast cancer sample using EMT gene signature (*Materials and Methods*) and correlated it with the level of PD-L1 [27]. We tested this correlation in GSE18864 dataset of breast cancer patients that was profiled on Affymetrix one-color arrays. Although the number of patients within this dataset was relatively small (*n* = 84), there was significant association between EMT status and PD-L1 expression (r = 0.24, p value = 0.026, Additional file 1: Figure S3A).

Previous findings showed significant correlation between PD-L1 expression and triple negative breast cancer (TNBC) [15], which has recently been further classified into different subtypes including basal-like and claudin-low. Among all subtypes, claudin-low breast cancer has the highest association with EMT status [5, 28, 29]. Therefore, we tested the correlation between PD-L1 expression with claudin-low subtype. Using the claudin-low gene signature, we calculated claudin-low score for each patient’s sample (as described in *Materials and Methods*). PD-L1 expression significantly correlated (r = 0.58, p value = 7.05x10^−9^) with claudin-low subtype (Additional file 1: Figure S3B). Among the 84 patients in GSE18864 dataset, 38 patients were TNBC. Interestingly, the statistical significance between EMT score and PD-L1 expression was higher (r = 0.42, p value = 0.0095) when analysis was limited to TNBC patients (Additional file 1: Figure S3C). Similarly, there was highly significant correlation between claudin-low and PD-L1 expression within TNBC patients (r = 0.73, p value = 2.65x10^−7^, Additional file 1: Figure S3D). These data show significant correlation between the PD-L1 expression level and the EMT status of breast cancer patients in the GSE18864 dataset.

**Fig. 4:**
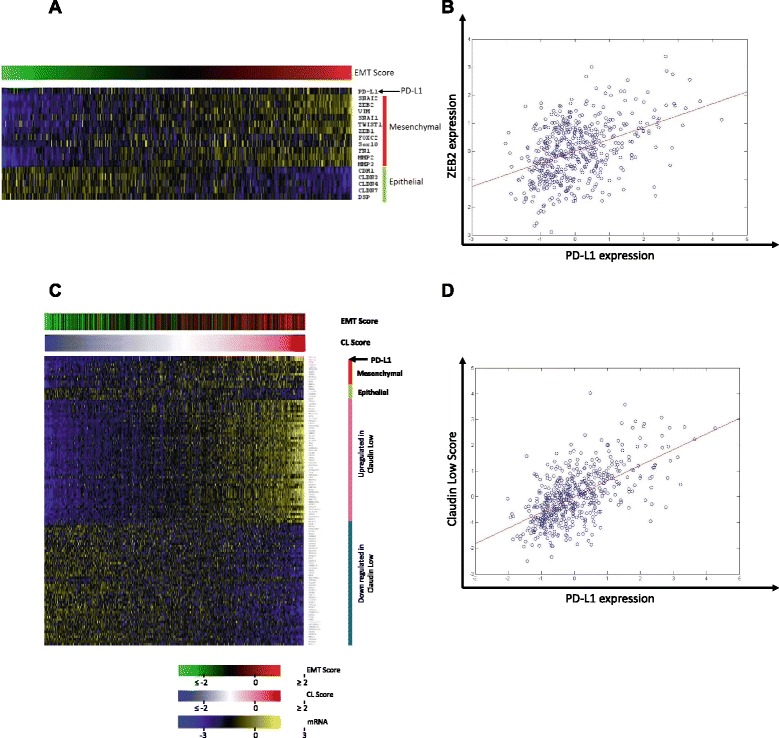
Correlation of PD-L1 expression, EMT and claudin-low subtype of breast cancer using TCGA gene expression dataset. Gene expression dataset from the TCGA breast cancer samples (total of 530 patients) showing the correlation of PD-L1 expression with EMT score and claudin-low breast cancer A&C). Heat map showing mRNA expression level of mesenchymal and Epithelial genes in addition to top 100 claudin-low gene expression signature (up- or down-regulated in CL). The samples in the data are arranged according to their EMT score (**a**) or according to their claudin-low score (**c**) and compared with PD-L1 expression, as indicated with the arrow at the top. B&D) Scatter plot of PD-L1 mRNA expression level correlates significantly that of with ZEB2 (**b**) or claudin-low score (**d**) in TCGA breast cancer samples (p < 0.0001)

We further used another well-known dataset, which uses a different array platform (Agilent) and with much larger patient size, the TCGA breast cancer dataset (*n* = 530 patients), to validate our findings on the association of PD-L1 expression with EMT status. In this large dataset there was a highly significant correlation (r = 0.14, *p* = 0.0009) between PD-L1 expression and EMT status (Fig. 4a). In fact, certain EMT inducing transcription factors (ZEB2, SNAI1, ZEB1) individually correlated with PD-L1 expression, with the most significant correlation (r = 0.42, *p* = 1.9x10^−24^) with ZEB2 (Fig. 4b). More interestingly, there was an extremely significant correlation (r = 0.61, *p* = 8x10^−54^) between claudin-low subset of breast cancer and PD-L1 expression level (Fig. 4 c&d). This demonstrates that PD-L1 is highly associated with EMT status and claudin-low subset of breast cancer in TCGA dataset of breast cancer patients.

Using multiple datasets of breast cancer patients, we have demonstrated that PD-L1 correlates with EMT status of breast cancer patients and molecular subtypes of breast cancer showing signs of EMT, further supports for the tight relationship between PD-L1 and EMT in breast cancer.

#### PD-L1 expression on the protein level is associated with EMT status of tumor cells in breast cancer patients

The above PD-L1 expression analysis on breast cancer patients was performed using mRNA expression, which does not always correlate with protein expression. Therefore, we tested the association between the PD-L1 protein level and EMT status in breast cancer patients using 67 breast cancer patients with previously known PD-L1 status [16]. The EMT status of breast cancer cells was assessed in by staining previously archived paraffin embedded sections with antibodies against vimentin and E-cadherin (Additional file 1: Figure S4A&B). PD-L1 expression significantly associated (*p* = 0.014) with vimentin expression in breast cancer cells (Table 1). Similarly, there was significant correlation with E-cadherin downregulation (*p* = 0.039). Interestingly, when the data is stratified to patients showing simultaneous vimentin upregulation and E-cadherin downregulation, the statistical correlation with PD-L1 expression was much more significant (*p* = 0.005). These findings demonstrate that the PD-L1 protein level correlates well with EMT status of tumor cells in breast cancer patients.

**Table 1:** Association between PD-L1 and markers of EMT in 67 breast cancer patients

	PD-L1 Expression		*P value
	+	-	
**E-Cadherin**			
Down-regulated (≤70 %)	9 (47)^a^	10 (53)	**0.039**
Positive (> 70 %)	10 (21)	38 (79)	
**Vimentin**			
Up-regulated (> 5 %)	10 (53)	9 (47)	**0.014**
Negative (<5 %)	9 (19)	39 (81)	
**E-Cadherin-down/Vimentin-up**			
Present	6 (75)	2(25)	**0.005**
Absent	13 (22)	46 (78)	

*Abbreviations*: (+ and -) are number of positive and negative patients, ^a^Numbers between brackets are the percentages of patients, *P values (by Fisher’s exact test) in bold represent a significant data

#### Manipulation of PD-L1 modulates EMT status of breast cancer cells

**Fig. 5:**
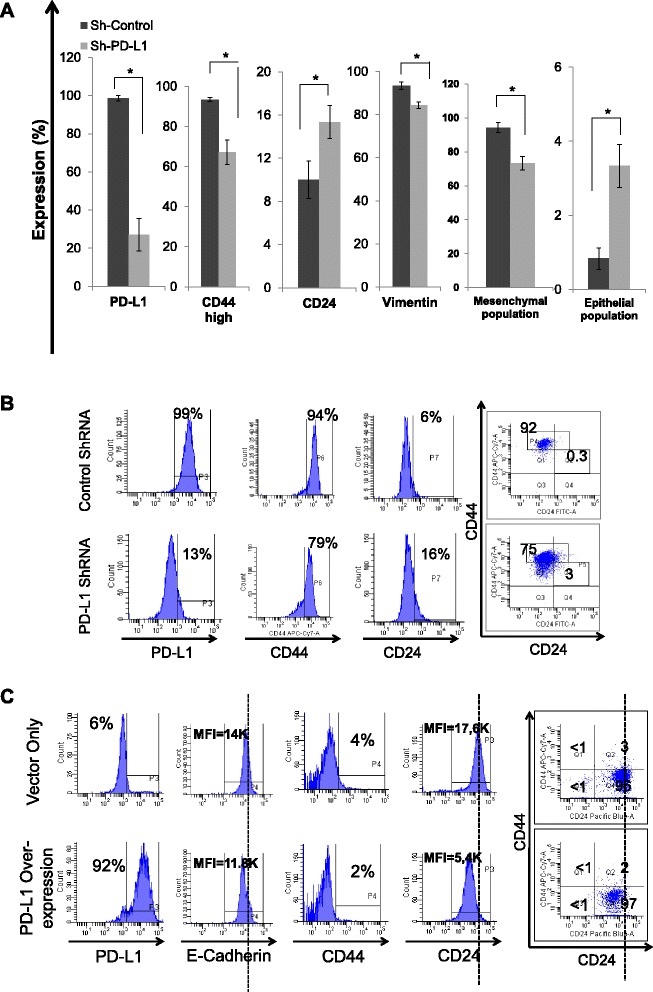
Manipulation of PD-L1 modulates EMT status of breast cancer cells. EMT status determined by the expression of CD44, CD24, and vimentin or CD44/CD24 combination as measured by flow cytometry following PD-L1 knockdown using specific Sh-RNA mesenchymal-like (MDA-MB-231) breast cancer cells (**a** & **b**). or PD-L1 overexpression by transfection with PD-L1 ORF in the luminal-like (T47D) breast cancer cells (**c**). Bars in **a** represent the means of 3 three different clones and 3 different experiments ± standard deviation (*n* = 9) while histograms in **b** & **c** are representative of one of the experiments. Lines in each histograms represent threshold of positivity as determined by isotype control except of CD44 in MDA-MB-231 cells where it is an arbitrary line to show CD44^high^ status

The above data show that PD-L1 is upregulated upon EMT and is predominantly expressed in claudin-low breast cancer. However, it is unknown if the PD-L1 molecule itself has any role in the EMT process. In order to address this, we have used specific ShRNA to downregulate PD-L1 in the claudin-low breast cancer cell line (MDA-MB-231), which expresses high level of PD-L1. Interestingly, downregulation of PD-L1 in MDA-MB-231 cells downregulated CD44 by 30 %, and vimentin by 10 % while it upregulated CD24 by 5 % (Fig. 5a & b). Using the combination of CD44 and CD24 shows that PD-L1 downregulation decreased the fraction of MDA-MB-231 cells with typical mesenchymal phenotype CD44^high^/CD24^low/neg^ and increased the rare epithelial-like fraction in MDA-MB-231 cells by up to 3 folds. We were able to confirm our findings using another different Sh-RNA that is under the control of doxycyline and we got similar results only when doxycycline was added confirming the specific effect of PD-L1 downregulation (Additional file 1: Figure S5). To further examine the role of PD-L1 on the EMT process in other subtypes of breast cancer we induced the expression of PD-L1 in T47D luminal-like breast cancer cell line which is typically negative for PD-L1. Overexpression of the PD-L1 molecule downregulated CD24 by 70 % and downregulated E-Cadherin expression by 16 %. However, there was only slight decrease in CD44 expression (Fig. 5c). Therefore, PD-L1 manipulation in breast cancer cells modulates the EMT status of these cells.

## Discussion

PD-L1 expression in breast cancer cells is significantly associated with hormone receptor negativity. However, the consequence of this association and the underlying mechanism has not been investigated before. In this study we have demonstrated that the pro-metastatic process EMT induces the expression of PD-L1. Furthermore, we have identified an association between PD-L1 expression and claudin-low breast cancer, a subset of breast cancer patients known to have high EMT score. Interestingly, we have shown that PD-L1 expression is important for the maintenance of the EMT status in this subtype of breast cancer cells.

EMT is a biological process whereby epithelial cells lose their cell-to-cell junctions and gain features of mesenchymal cells like higher migration, and invasion. This process is paralleled by a change in some of the markers like CD44, CD24, vimentin and E-cadherin. Mesenchymal cells have a CD44^high^/CD24^low^, vimentin^pos^ and E-cadherin^neg^ phenotype, while epithelial cells normally have CD24^high^, vimentin^neg^ and E-Cadherin^pos^ phenotype. During the EMT process epithelial cells acquire the CD44^high^/CD24^low^ phenotype, up-regulate vimentin and down-regulate E-Cadherin.

We and others have reported several mechanisms that regulate PD-L1 expression in breast cancer cells. These include the presence of inflammatory mediators within the tumor microenvironment [17], the loss of phosphatase and tensin homologue (PTEN), a negative regulator of PI3K activation [18], and cancer cell proliferation [19]. However, none of the previously reported mechanisms explained the constitutive expression of PD-L1 in cancer cells that lacks PTEN mutation/deletion and in the absence of inflammatory mediators. We have shown here that the EMT process induces PD-L1 expression and that the expression of this molecule participates in the maintenance of EMT status in the breast cancer cells.

The main signaling pathways involved in the EMT process are PI3K/AKT, SMAD, NF-κB and ERK/MAPK pathways (Reviewed in [25]). It is believed that the different features of EMT involve different pathways. For example, PI3K/AKT is important for the EMT-related enhanced migration of cells [30], while NF-κB is involved in the EMT induced chemoresistance of tumor cells [31]. Importantly, we have shown that EMT-induced upregulation of PD-L1 is PI3K/AKT-and ERK/MEK-dependent. Although both pathways were important for the up-regulation of PD-L1, the involvement of PI3K/AKT pathway was more important. This finding is consistent with previous findings showing critical role of the PI3K/AKT pathway in the expression of PD-L1 in breast cancer [32]. Similarly, the involvement of the ERK/MEK pathway in PD-L1 expression has been previously reported [33].

The induction of EMT in breast cancer cells is pathologically relevant. It is highly associated with claudin-low breast cancer which has received a lot of attention recently. Interestingly, this subset of breast cancer is associated with a high degree of immune cell infiltrate and bad prognosis, suggesting the presence of an ineffective anti tumor immunity. The exact immune suppressive molecules associated with this subset of breast cancer is not well-described. Interestingly, induction of EMT in breast cancer cells using Snail, an EMT transcription factor, promoted the escape of breast cancer cells from T-cell mediated lysis by CD8+ cytotoxic T cells [34]. This finding supports the role of EMT in the immune escape of breast cancer cells. We have shown for the first time that claudin-low subtype of breast cancer is associated with high PD-L1 expression, which is likely due to EMT, and that can lead to immune suppression and/or activation of an ineffective immune response. During the preparation of this manuscript, a novel data emerged showing similar findings in lung cancer, suggesting that our novel finding in breast cancer is a broad phenomena that might include many carcinomas [27].

Recently, a targeted therapy for PD-L1 became available and it has shown great success in several types of carcinomas like non-small cell lung cancer and melanoma. These trials are encouraging to try this therapy against breast carcinomas. However, it is important to choose the appropriate patients who would likely benefit from this treatment. Our findings suggest that claudin-low breast cancer patients who express high level of this gene, have EMT and thus possibly can benefit from the reversal of the immune process into a more favorable cytotoxic type. Recent data showed that patients who would likely benefit from PD-L1 therapy typically have tumor infiltrated with exhausted CD8+ T-cells and overexpress PD-L1 [35, 36]. Given that claudin-low breast cancer is associated with high expression of PD-L1 and immune cell infiltration, PD-L1 blockade will likely reverse the immune suppression and subsequently leads to activation of anti-tumor activity.

Importantly, there is an established link between EMT and the gain of stem cell functions in breast cancer [37]. These functions include the ability to grow in anchorage independent conditions and to reestablish tumors in mice models even when a very small number of cells are transplanted. Therefore, it is possible that by targeting PD-L1 we can target these cells, which constitute the main seed for tumor initiation and maintenance. To this end, further investigations are required.

## Conclusion

In conclusion the present data show that EMT status of breast cancer cells is an important regulator of PD-L1 constitutive expression in breast cancer cells. In addition, we have shown that PD-L1 expression is tightly associated with claudin-low breast cancer and that targeting PD-L1 expression would reverse EMT in this type of cells.

## Additional file

Additional file 1: Figure S1. Gating strategy to analyze PD-L1 expression in breast cells using multicolor flow cytometry (9 colors). Single isolated cell from normal breast tissue obtained from reduction mammoplasty were analyzed with sequential gating. Forward scatter (FSC) and side scatter (SSC) to extract cells from debris. DAPI positive cells were excluded to gate on viable cells only. CD45 were used to exclude hematopoietic cells followed by gating on PD-L1 positive cells. PD-L1 positive cells were further analyzed for Ep-CAM/CD49f, CD44/CD24 and CD90/CD31 expression levels. **Figure S2**. Upregulation of PD-L1 upon EMT induction in both normal and breast cancer cells. A) Flow cytometry histogram showing the expression level of PD-L1 molecule before and after 3 days of continuous TGF-β1 treatment in normal (MCF10A and MCF12A) and breast cancer (SK-BR-3, Hs578T and BT-594) cells. B) PD-L1 expression before and after EMT induction by transfection with H-RAS in MCF-12A cells as measured by flow cytometry. **Figure S3**. PD-L1 expression (mRNA) in breast cancer dataset correlated with EMT score and Claudin-low breast cancer. Gene expression dataset from the GSE18864 (total of 84 patients) showing the correlation of PD-L1 expression with EMT score (A&C) and Claudin-low breast cancer subtype (B&D). Heatmap shows mRNA expression level of mesenchymal and epithelial genes (A&C) in addition to top 100 (up- or down-regulated) claudin-low gene signature (B&D) from patients arranged according to their EMT score (A&C) or claudin-low score (B&D). The data in A&B are from the whole GSE18864 dataset (*n* = 84) while in C&D it is limited to TNBC patients (*n* = 38) only. **Figure S4**. PD-L1 expression correlates with Vimentin upregulation and E-cadherin downregulation. A) Representative images of paraffin embedded section of breast cancer stained with E-cadherin or vimentin and examined under light microscope B) Representative images of normal duct in one of these sections stained with E-cadherin or vimentin showing typical staining pattern. (PPTX 2880 kb)

## Abbreviations

PD-L1: Programmed death ligand-1
EMT: Epithelial to mesenchymal transition
CSCs: Cancer stem cell
