# Supplementary material for: Bidirectional crosstalk between PD-L1 expression and epithelial to mesenchymal transition: Significance in claudin-low breast cancer cells
Source: Mol Cancer. 2015 Aug 7;14:149. doi: 10.1186/s12943-015-0421-2 (PMC4527106; doi:10.1186/s12943-015-0421-2)
Supplement: Additional file 1: Figure S1. — Gating strategy to analyze PD-L1 expression in breast cells using multicolor flow cytometry (9 colors). Single isolated cell from normal breast tissue obtained from reduction mammoplasty were analyzed with sequential gating. Forward scatter (FSC) and side scatter (SSC) to extract cells from debris. DAPI positive cells were excluded to gate on viable cells only. CD45 were used to exclude hematopoietic cells followed by gating on PD-L1 positive cells. PD-L1 positive cells were further analyzed for Ep-CAM/CD49f, CD44/CD24 and CD90/CD31 expression levels. Figure S2. Upregulation of PD-L1 upon EMT induction in both normal and breast cancer cells. A) Flow cytometry histogram showing the expression level of PD-L1 molecule before and after 3 days of continuous TGF-β1 treatment in normal (MCF10A and MCF12A) and breast cancer (SK-BR-3, Hs578T and BT-594) cells. B) PD-L1 expression before and after EMT induction by transfection with H-RAS in MCF-12A cells as measured by flow cytometry. Figure S3. PD-L1 expression (mRNA) in breast cancer dataset correlated with EMT score and Claudin-low breast cancer. Gene expression dataset from the GSE18864 (total of 84 patients) showing the correlation of PD-L1 expression with EMT score (A&C) and Claudin-low breast cancer subtype (B&D). Heatmap shows mRNA expression level of mesenchymal and epithelial genes (A&C) in addition to top 100 (up- or down-regulated) claudin-low gene signature (B&D) from patients arranged according to their EMT score (A&C) or claudin-low score (B&D). The data in A&B are from the whole GSE18864 dataset (n = 84) while in C&D it is limited to TNBC patients (n = 38) only. Figure S4. PD-L1 expression correlates with Vimentin upregulation and E-cadherin downregulation. A) Representative images of paraffin embedded section of breast cancer stained with E-cadherin or vimentin and examined under light microscope B) Representative images of normal duct in one of these sections stained with E-cadherin or vimentin showing typic [file 12943_2015_421_MOESM1_ESM.pptx]

## Slide 1
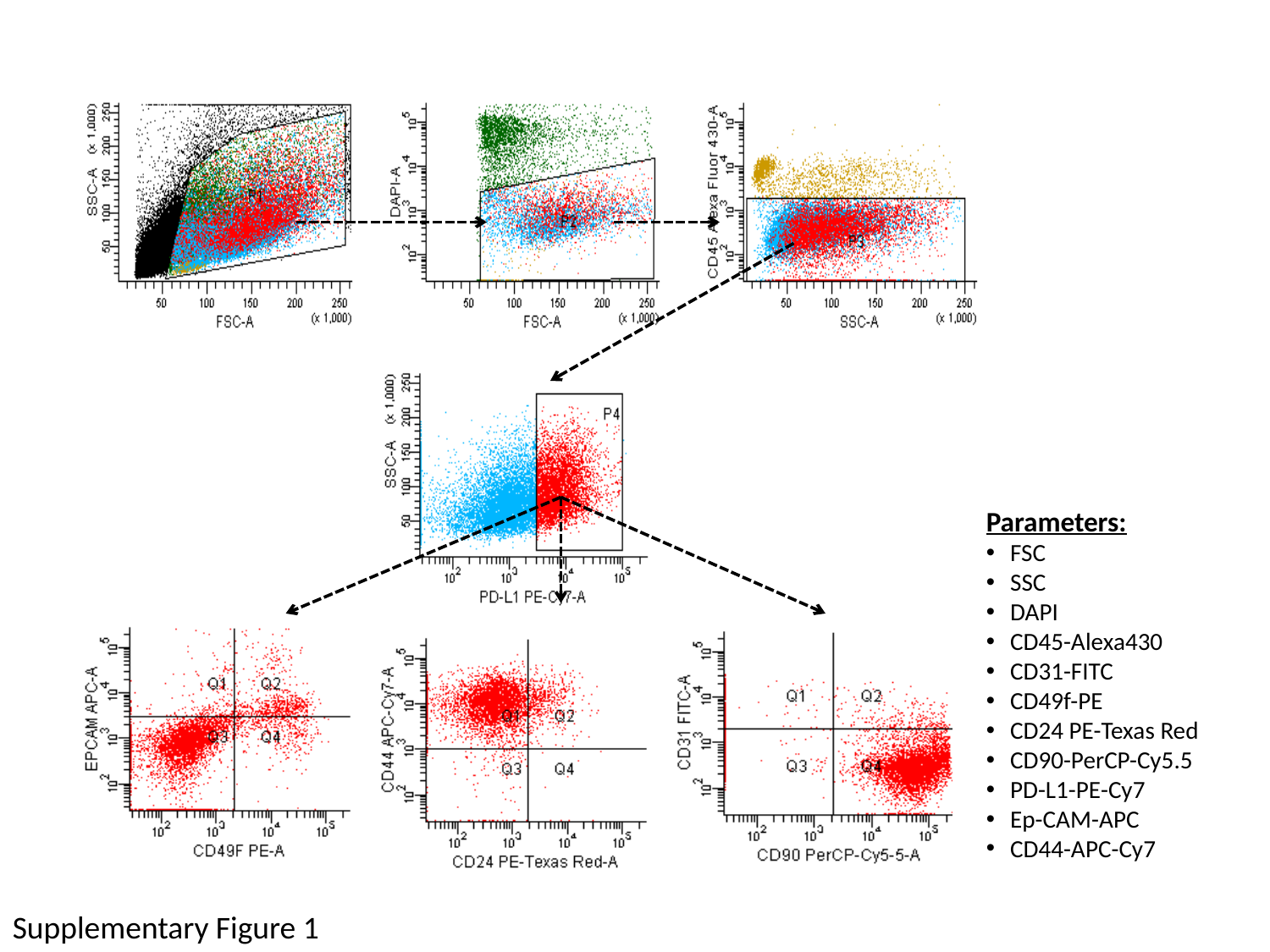

Parameters:
FSC
SSC
DAPI
CD45-Alexa430
CD31-FITC
CD49f-PE
CD24 PE-Texas Red
CD90-PerCP-Cy5.5
PD-L1-PE-Cy7
Ep-CAM-APC
CD44-APC-Cy7
Supplementary Figure 1

## Slide 2
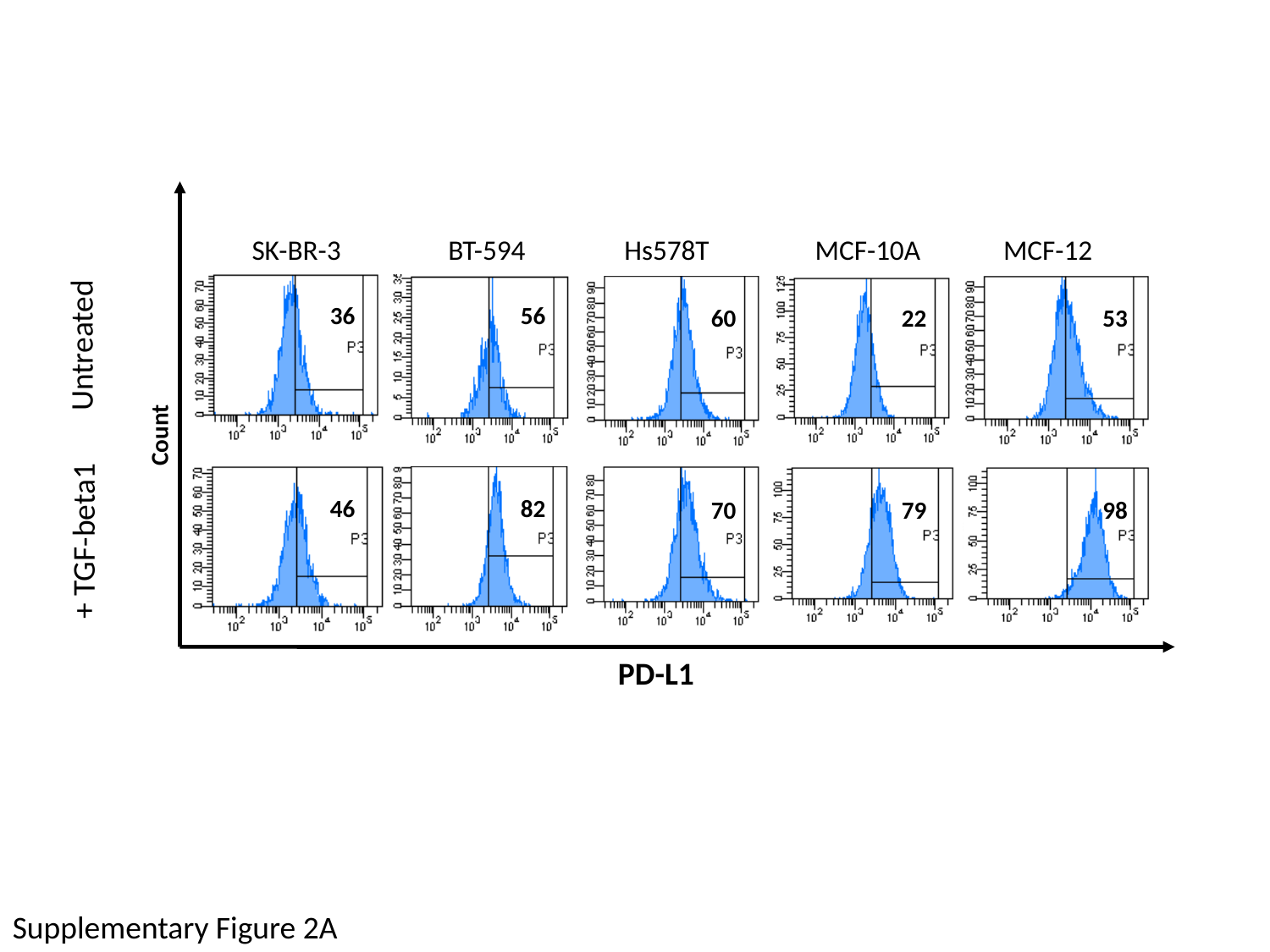

SK-BR-3
BT-594
Hs578T
MCF-10A
MCF-12
36
56
60
22
53
Untreated
Count
46
82
70
79
98
+ TGF-beta1
PD-L1
Supplementary Figure 2A

## Slide 3
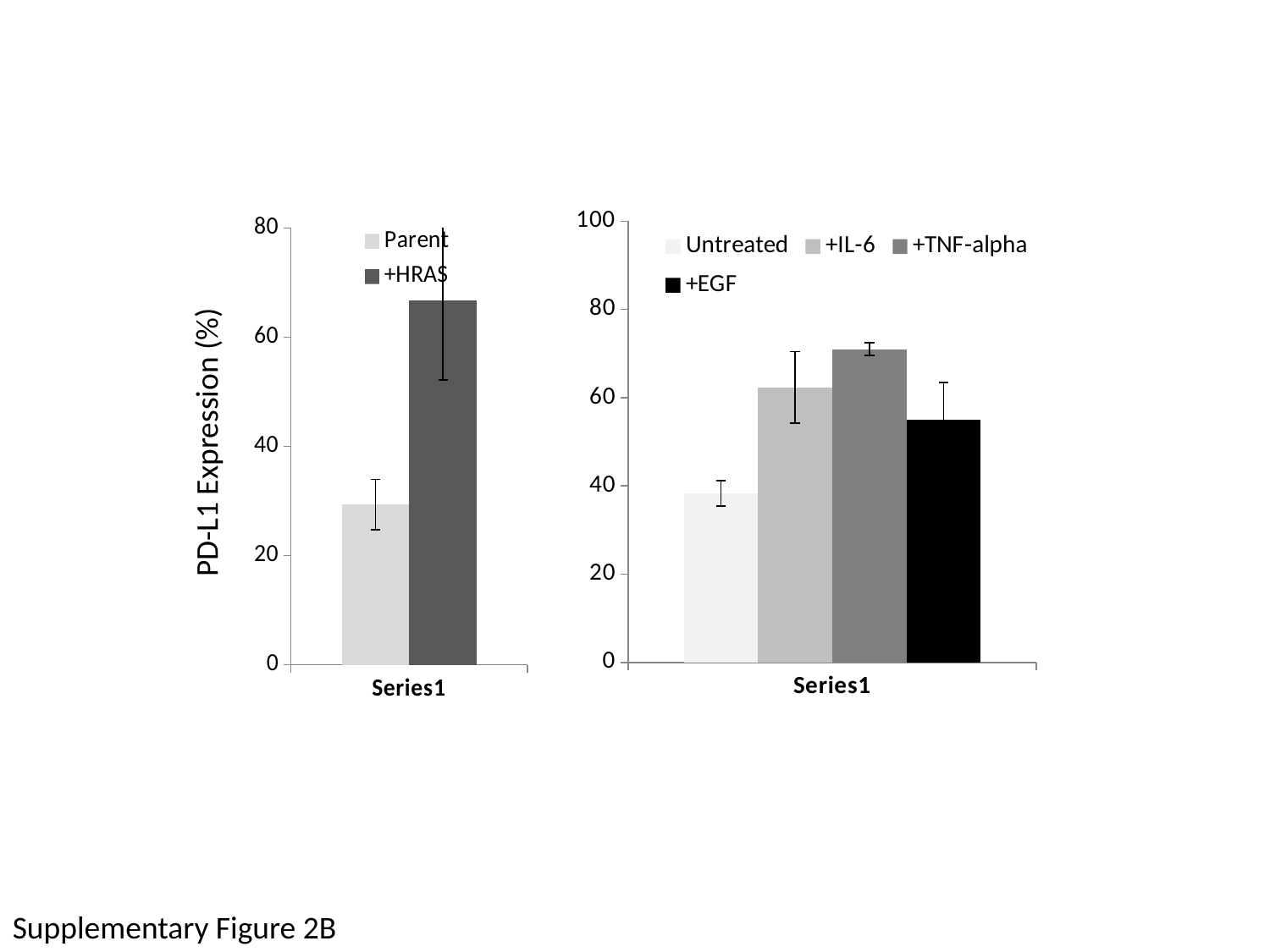

### Chart
| Category | Untreated | +IL-6 | +TNF-alpha | +EGF |
|---|---|---|---|---|
| | 38.333333333333336 | 62.333333333333336 | 71.0 | 55.0 |
### Chart
| Category | Parent | +HRAS |
|---|---|---|
| | 29.333333333333282 | 66.66666666666667 |PD-L1 Expression (%)
Supplementary Figure 2B

## Slide 4
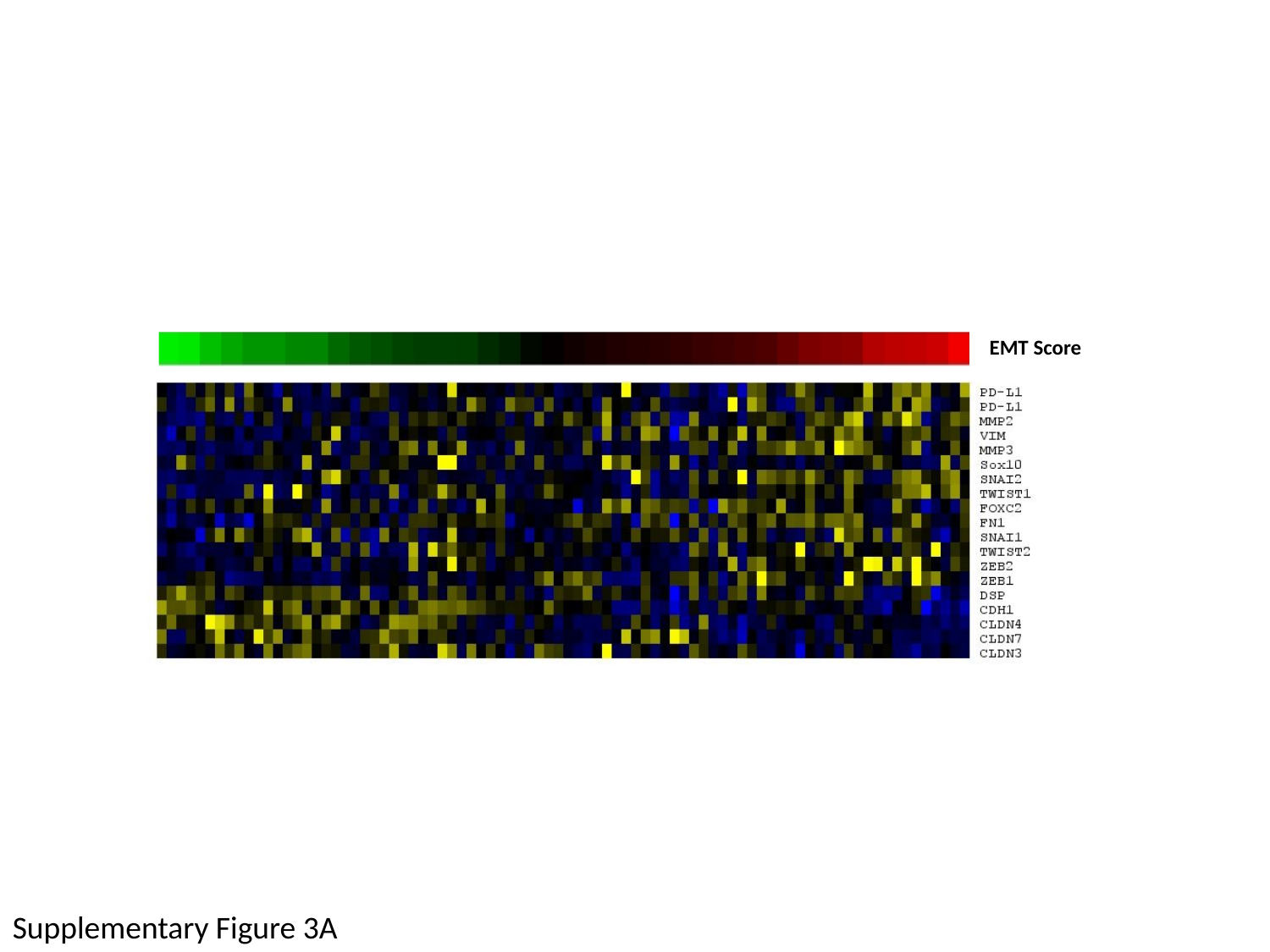

EMT Score
Supplementary Figure 3A

## Slide 5
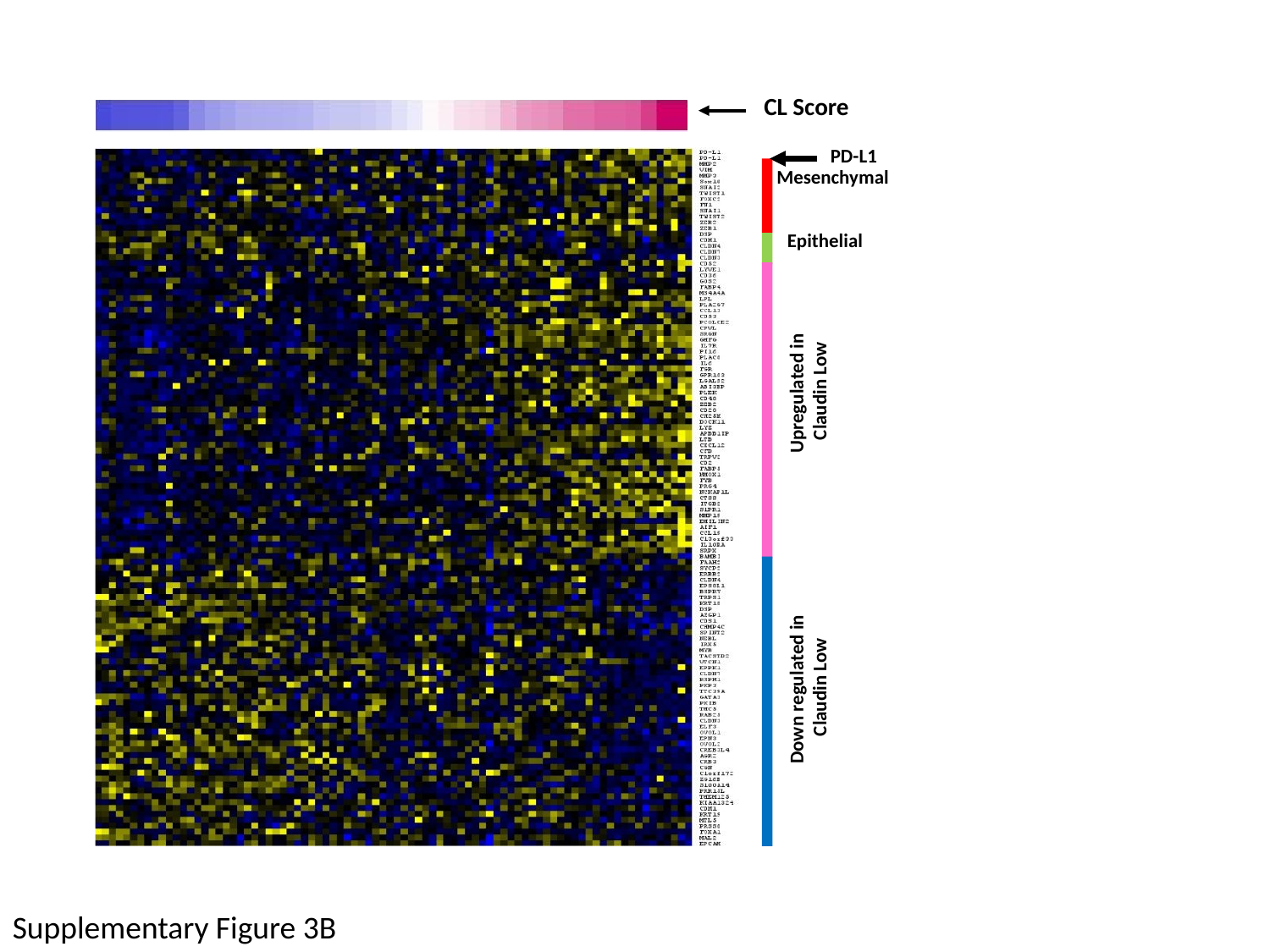

CL Score
PD-L1
Mesenchymal
Epithelial
Upregulated in
Claudin Low
Down regulated in
Claudin Low
Supplementary Figure 3B

## Slide 6
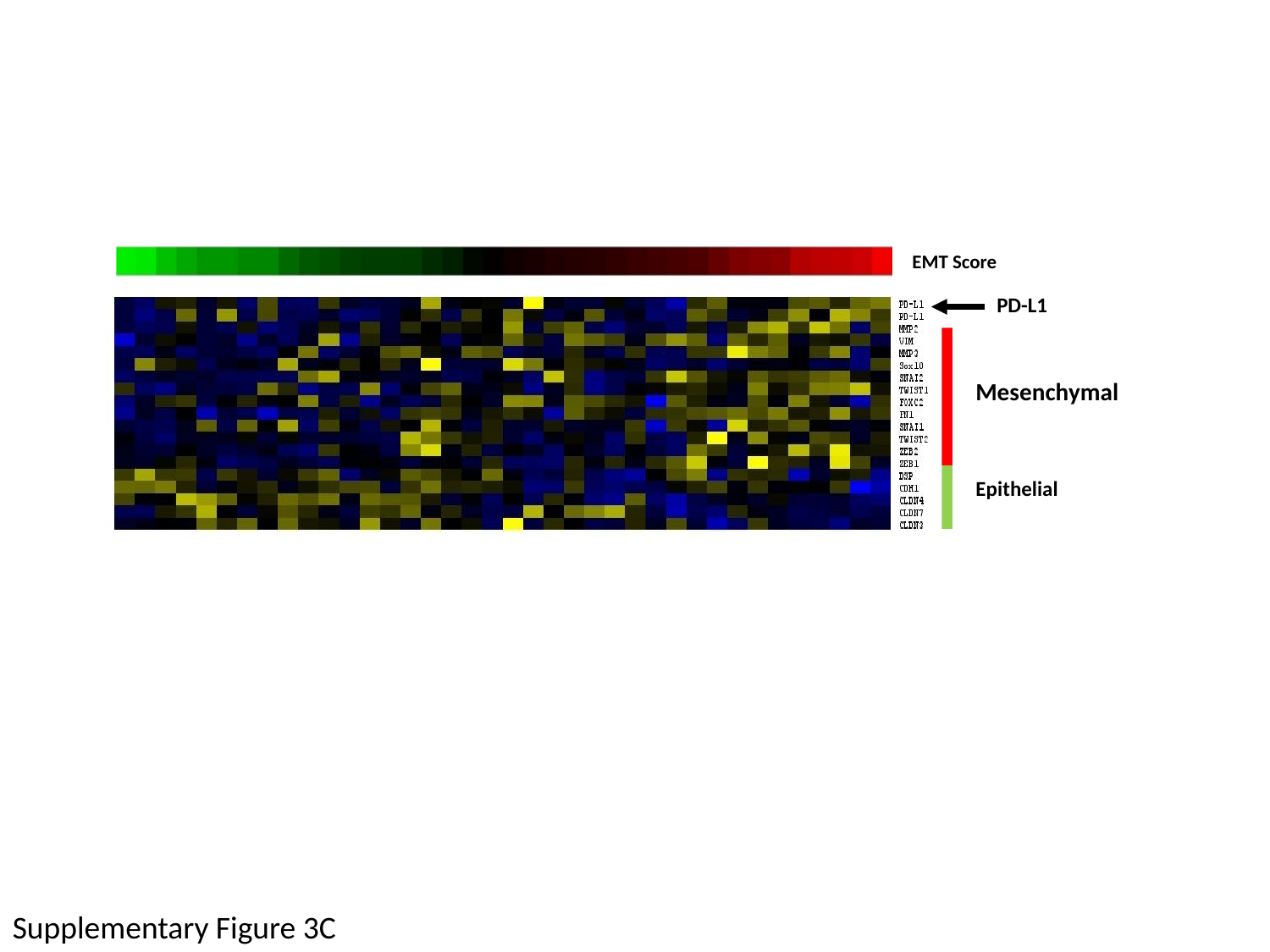

EMT Score
PD-L1
Mesenchymal
Epithelial
Supplementary Figure 3C

## Slide 7
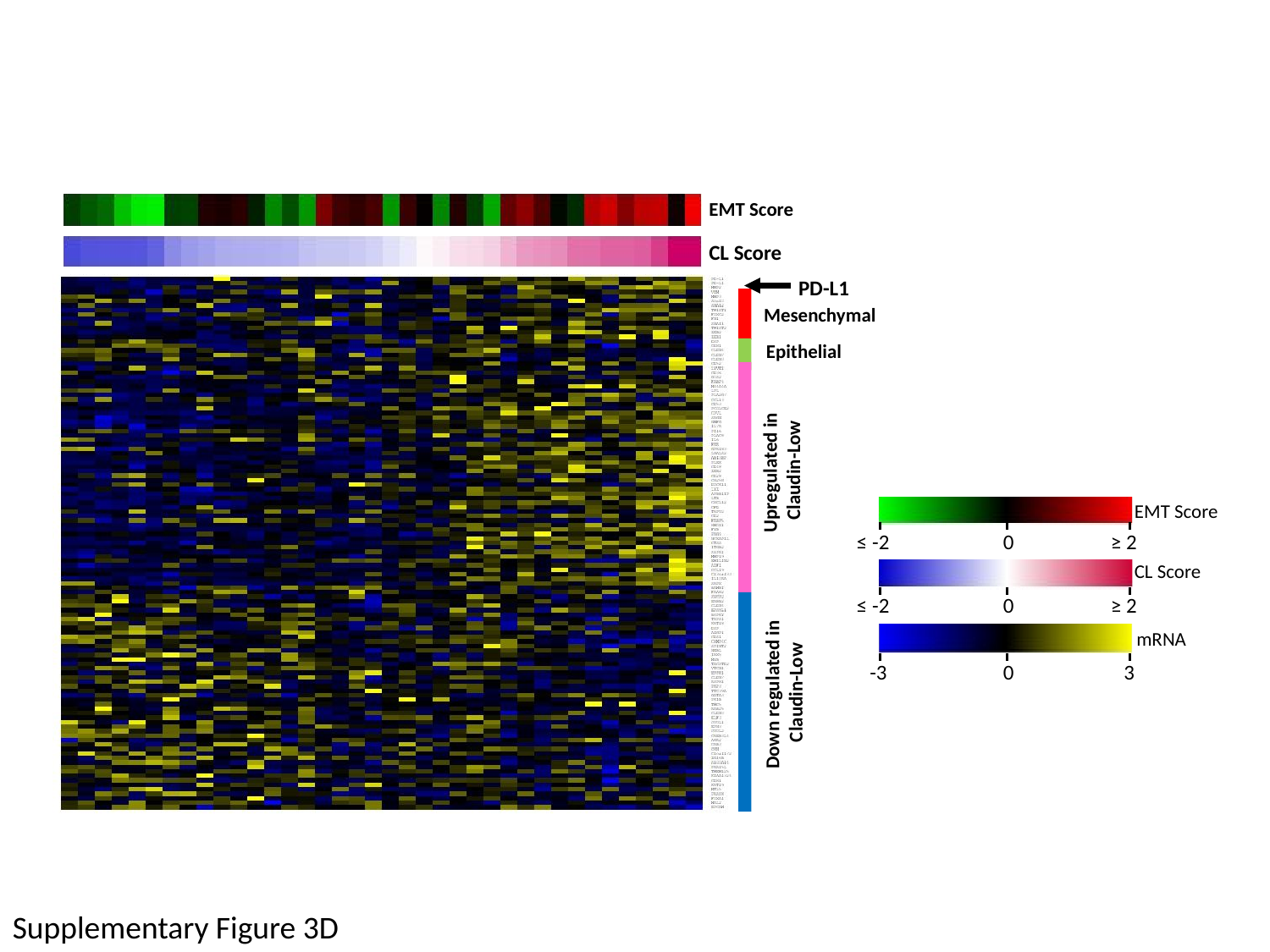

EMT Score
CL Score
PD-L1
Mesenchymal
Epithelial
Upregulated in
Claudin-Low
EMT Score
≤ -2
≥ 2
0
CL Score
≥ 2
≤ -2
0
mRNA
-3
0
3
Down regulated in
Claudin-Low
Supplementary Figure 3D

## Slide 8
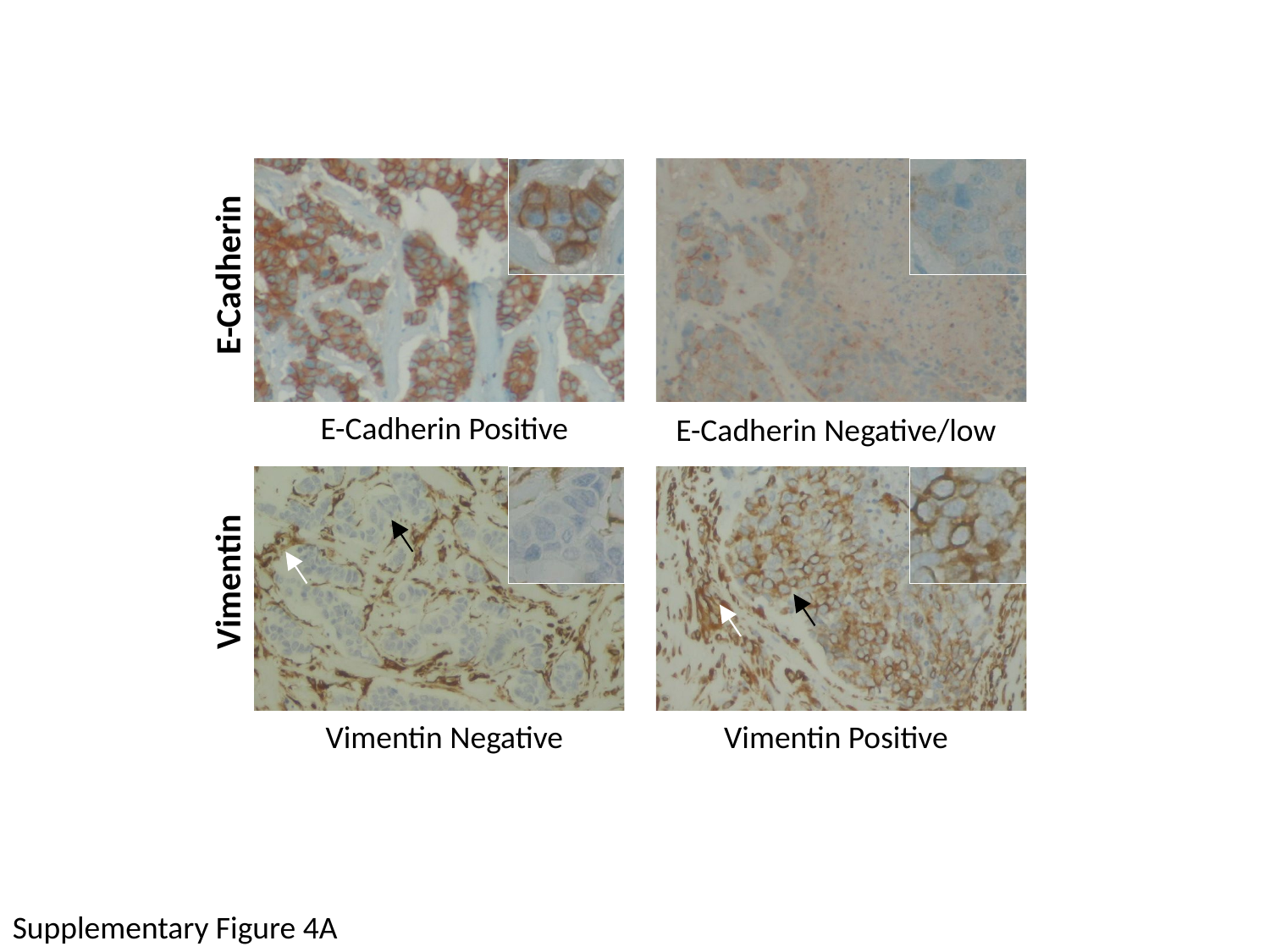

E-Cadherin Positive
E-Cadherin Negative/low
E-Cadherin
Vimentin Negative
Vimentin Positive
Vimentin
Supplementary Figure 4A

## Slide 9
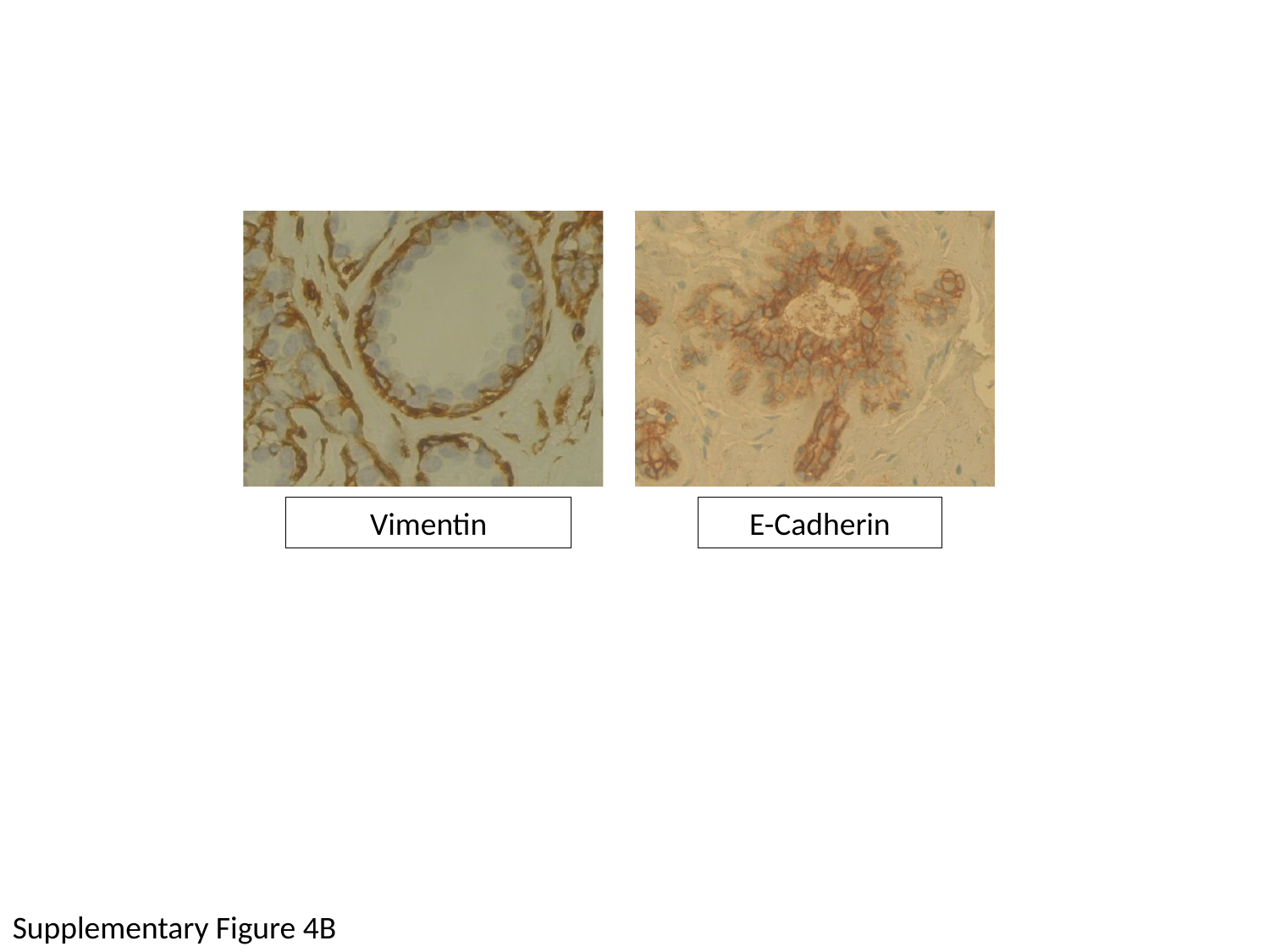

Vimentin
E-Cadherin
Supplementary Figure 4B

## Slide 10
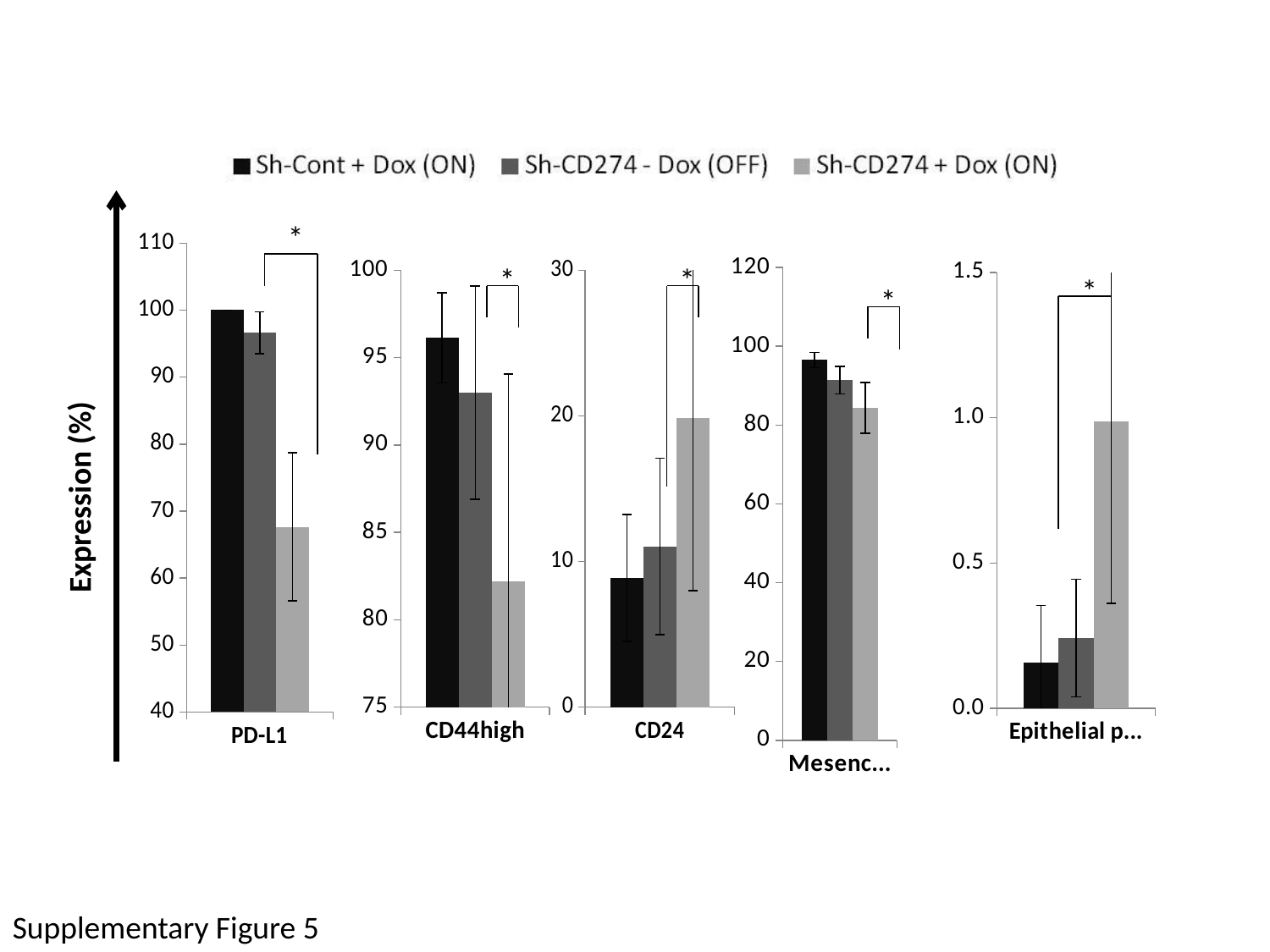

*
### Chart
| Category | Sh-Cont + Dox (ON) | Sh-CD274 - Dox (OFF) | Sh-CD274 + Dox (ON) |
|---|---|---|---|
| PD-L1 | 100.0 | 96.62499999999999 | 67.642857142857 |
### Chart
| Category | Sh-Cont + Dox (ON) | Sh-CD274 - Dox (OFF) | Sh-CD274 + Dox (ON) |
|---|---|---|---|
| CD44high | 96.12499999999999 | 93.0 | 82.21428571428572 |
### Chart
| Category | Sh-Cont + Dox (ON) | Sh-CD274 - Dox (OFF) | Sh-CD274 + Dox (ON) |
|---|---|---|---|
| CD24 | 8.87500000000002 | 11.023076923076925 | 19.835714285714282 |
### Chart
| Category | Sh-Cont + Dox (ON) | Sh-CD274 - Dox (OFF) | Sh-CD274 + Dox (ON) |
|---|---|---|---|
| Mesenchymal population | 96.5 | 91.38461538461554 | 84.35714285714282 |
### Chart
| Category | Sh-Cont + Dox (ON) | Sh-CD274 - Dox (OFF) | Sh-CD274 + Dox (ON) |
|---|---|---|---|
| Epithelial population | 0.15714285714285744 | 0.24166666666666672 | 0.9874999999999995 |*
*
*
*
Expression (%)
Supplementary Figure 5
